# Electrical Muscle Stimulation with Russian Current in Chronic Cerebral Ischaemia †

**DOI:** 10.3390/life16010126

**Published:** 2026-01-14

**Authors:** Nelly M. A. Artamonova, Alina A. Saveko, Tatiana A. Shigueva, Vladimir V. Kitov, Maria A. Avdeeva, Valentina N. Tsyganova, Tatyana Yu. Orestova, Alla B. Guekht, Elena S. Tomilovskaya

**Affiliations:** 1 Russian Federation State Research Center, Institute of Biomedical Problems RAS (IBMP), 123007 Moscow, Russiat.shigueva@gmail.com (T.A.S.); finegold@yandex.ru (E.S.T.); 2 Research and Clinical Center for Neuropsychiatry, Moscow Healthcare Department, 127006 Moscow, Russiainfo@npcpn.ru (T.Y.O.);; 3 Department of Neurology, Neurosurgery and Medical Genetics, Pirogov Russian National Research Medical University, 117997 Moscow, Russia

**Keywords:** Russian current, gait, balance, rehabilitation, chronic cerebral ischaemia, electrical muscle stimulation, stabilography, timed up and go test, Tinetti test

## Abstract

Objective: To test whether inpatient electrical muscle stimulation (EMS) using Russian current (5 kHz carrier, 50 Hz modulation; 4 s ON/6 s OFF) improves mobility and balance in elderly people with chronic cerebral ischaemia. Design: Prospective single-centre controlled observational pilot, embedded in routine inpatient rehabilitation; no concealed randomisation (EMS + standard care; sham EMS + standard care; standard care only (control)). Methods: A single-centre controlled observational study with three groups was conducted (EMS n = 27, control n = 10, sham n = 7) with 3–9 sessions over 2 weeks (20 min; quadriceps and calves). Pre/Post Outcomes: Tinetti (balance/gait), Rivermead Mobility Index, Timed Up and Go (TUG), ankle extensor maximal voluntary force (MVF), stabilography (statokinesiogram path length (L), mean velocity of COP (V), sway area (S), and myotonometry; ANOVA, α = 0.05). Ethics approval and informed consent were obtained. Between-group differences in change scores were evaluated descriptively, and no formal hypothesis-testing was planned. Results: EMS showed significant gains versus control/sham—higher Tinetti total and Rivermead scores, faster TUG, higher MVF, and improved stabilography in the eyes-closed condition (reduced L, V, and S), with good tolerability and no serious adverse events (SAEs). Conclusions: Short-course Russian-current EMS is feasible and associated with clinically meaningful improvements in balance, gait, and strength in elderly patients with chronic cerebral ischaemia; however, larger randomised trials are warranted.

## 1. Introduction

Physical inactivity is common among elderly patients and is both a cause and a consequence of decreased quality of life and disability. The effects of ageing, particularly a sedentary lifestyle, are a significant cause of loss of contractility and skeletal muscle mass in older adults [1,2]. These patients are not always able to maintain a sufficient level of physical activity. Stroke and chronic cerebral ischaemia (ICD-10 I67.8) are leading causes of disability worldwide; persistent gait impairment, reduced postural stability, and weakness of knee extensors and ankle musculature drive dependence and elevate fall risk in this population. Despite progress in task-specific rehabilitation, many patients plateau with clinically important residual deficits. Adjunctive modalities capable of safely increasing force output, reinforcing functional motor patterns, and expanding tolerable practice volume remain a priority in inpatient and early post-acute care. This study hypothesised that EMS using the so-called Russian current, which has demonstrated prophylactic efficacy in cosmonauts, may also be effective for patients with chronic cerebral ischaemia (CCI) with impaired motor function [3,4].

“Russian current” (RC) denotes a medium-frequency sinusoidal alternating current (typically 2.5–5 kHz) delivered in bursts around ~50 Hz to elicit fused (tetanic) contractions while maintaining an acceptable cutaneous sensation. The approach is historically attributed to Yakov M. Kots and colleagues, whose early studies popularised the label “Russian current” in the sports and rehabilitation literature [5,6]. Subsequent methodological syntheses clarified that the defining features of RC are the sinusoidal carrier in the kilohertz range and burst modulation near 50 Hz rather than any single proprietary waveform or device; these features were codified in foundational reviews and remain the basis for contemporary protocols and textbooks [7]. In this study, we use the term electrical muscle stimulation (EMS) to denote our Russian-current EMS protocol applied to lower-limb muscles, whereas EMS refers to the broader class of stimulation techniques evaluated in systematic reviews.

Two properties of RC are clinically salient. First, increasing the carrier frequency into the kilohertz range reduces skin impedance, often allowing higher comfortable currents at the skin–electrode interface for a given perceived intensity; this can be advantageous in older patients who report discomfort with conventional low-frequency pulses [5,7]. Second, burst rates around 50 Hz lie within the tetanus-producing range for human skeletal muscle, enabling neuromuscular re-education and progressive strengthening when work–rest cycles are well-chosen to mitigate early fatigue. Theoretically, RC can preferentially recruit higher-threshold motor units when voluntary drive is insufficient—an effect relevant for deconditioned or selectively denervated muscles after stroke—while tolerability ensures the session duration is adequate for clinically meaningful practice.

The empirical basis for EMS after stroke is substantial: systematic reviews and meta-analyses report improvements in gait, speed, and balance measures when stimulation is integrated into structured programs [8,9,10]. Across the broader literature, RC-specific clinical data are emerging. A pilot randomised controlled trial in chronic stroke survivors applying quadriceps RC in addition to usual care reported significant gains in 10-m walk speed, with no serious adverse events (SAEs), supporting the feasibility and potential functional benefit of RC in lower-limb motor recovery [11]. Together, these strands suggest that RC can be a tolerable, parameter-sensitive option for eliciting functionally relevant contractions during task-oriented therapy, while also emphasising the need for complete and transparent reporting to enable replication. The electrical myostimulation approach to reducing these risks does not require vigorous physical activity, which is difficult for this group of patients, and is also a method that can be applied during hospitalisation [12,13]. In this case, EMS can be an alternative to active physical training [14]. This method is preferred due to its good patient tolerability and minimal pain [15].

Heterogeneity of reporting has historically limited synthesis across RC studies. Methodological sources highlight several elements that should be prespecified: (I) carrier frequency (commonly 2.5–5 kHz); (II) burst frequency (~50 Hz); (III) duty cycle and work–rest timing (e.g., 4 s ON/6 s OFF or 10 s/10 s); (IV) intensity titration reported in mA and as percent of maximally tolerated current (%MTC) with safety stop rules; and (V) electrode placement over motor points, ideally using validated atlases and palpation-based verification [16]. Inadequate specification of these factors can obscure dose–response relationships, confound comparative interpretation, and limit clinical translation.

Clinical reasoning suggests several scenarios in which RC may be particularly useful: (a) patients with sensory hypersensitivity or fragile skin, for whom the sensation produced by RC is better tolerated at functional intensities; (b) patients requiring higher delivered currents to achieve visible, task-relevant contractions within constrained therapy time; and (c) gait and balance programs that emphasise ankle strategy and sit-to-stand transitions, where strengthening the triceps surae and quadriceps femoris is directly linked to functional endpoints (Timed Up and Go (TUG) and Tinetti subscales). Compared with conventional low-frequency neuromuscular electrical stimulation, Russian-current EMS uses a kilohertz carrier that reduces skin impedance and is often perceived as more comfortable and better tolerated, allowing the delivery of higher stimulation intensities in older patients [7,17,18]. In such contexts, short ON periods and built-in OFF intervals can manage early fatigue in deconditioned musculature while preserving the neuromuscular benefits of repetitive, task-integrated contractions.

The primary aim of this prospective single-centre controlled observational pilot was to evaluate the feasibility, tolerability, and short-term within-group functional changes associated with adding Russian-current EMS to routine inpatient rehabilitation for older adults with CCI and impaired gait and balance. We hypothesised that EMS would be feasible to deliver in this setting and would be associated with within-group improvements in balance, mobility, ankle extensor strength, and postural stability over approximately 2 weeks. Given the non-random allocation and modest sample size, between-group differences in change were prespecified as descriptive.

## 2. Materials and Methods

### 2.1. Study Design

This was a prospective, single-centre, controlled observational pilot embedded in routine inpatient neurorehabilitation at the Research and Clinical Center for Neuropsychiatry of the Moscow Healthcare Department (Moscow, Russia) from September 2021 to October 2024. Patients were managed within the existing inpatient pathway for stroke and chronic cerebral ischaemia, and were allocated in routine practice to one of three groups:

(1) EMS + Standard Care: Russian-current EMS applied to lower-limb muscles in addition to the standard rehabilitation program.
(2) Sham EMS + Standard Care: Sham stimulation with identical electrode placement and timing but sub-motor sensory-level intensity, plus standard rehabilitation.
(3) Standard Care Only (Control): Standard rehabilitation without EMS.

There was no concealed randomisation. Allocation reflected routine clinical decision-making by the attending neurologist. In broad terms, patients were considered for EMS if they met the general inclusion criteria, had no contraindications to electrical stimulation (e.g., unstable arrhythmia, uncontrolled epilepsy, significant skin lesions under electrodes), and were judged able to cooperate with the procedures. Some patients were managed with standard care alone because of relative contraindications, concerns about tolerance, or clear preference for non-stimulation. The sham group comprised patients for whom EMS was deemed appropriate but who, for organisational reasons, were scheduled with low-intensity sensory-level stimulation mimicking active EMS. Although the same inclusion and exclusion criteria were applied across groups, the resulting group sizes were unequal (EMS n = 27; control n = 10; sham n = 7), which affects interpretability and increases the potential for confounding.

This study followed the Declaration of Helsinki and national regulations, and was approved by the local bioethics committee (Protocol No. 49: 30 July 2021). All participants provided written informed consent. Reporting follows the STROBE recommendations for observational studies, and a completed STROBE checklist is provided in Supplementary Table S1.

### 2.2. Participants

This study included fifty-two inpatients aged 60–90 years with chronic cerebral ischaemia and impaired gait and balance; eight patients discontinued participation prematurely. Participants were divided into three groups as described above: the EMS + standard care group (27 patients), the standard-care-only (control) group without stimulation (10 patients), and the sham EMS + standard care group (7 patients). Baseline demographic characteristics (age, sex, height, weight, Mini-Mental State Examination (MMSE) score) are summarised in Table 1, which is included in the manuscript. Briefly, the mean age in the EMS, control, and sham groups was 72.96 ± 7.34, 79.0 ± 7.0, and 71.22 ± 8.30 years, respectively. Mean MMSE scores were 27.62 ± 1.47, 26.67 ± 1.23, and 27.78 ± 1.20 points, respectively, indicating generally preserved global cognition. Inclusion criteria were as follows: aged 60–90 years; impaired gait and balance functions in patients with chronic cerebral ischaemia, coded in our national ICD-10-based system as I67.8 (“Other specified cerebrovascular diseases”)—typically reflecting chronic cerebrovascular insufficiency due to atherosclerosis and arterial hypertension—confirmed by clinical examination and validated tests; ability to follow simple instructions and participate in the rehabilitation program. Baseline demographic and clinical characteristics of the three study groups, including age, sex, and cognitive status, are presented in Table 1. Exclusion criteria were as follows: high spasticity in the lower limbs (score ≥ 3 on the Modified Ashworth Scale); atrial fibrillation or other significant cardiac rhythm disturbances; active infectious processes, wound surfaces, or skin irritation at electrode placement sites; epilepsy; severe orthopaedic pathology (e.g., total arthroplasty of major lower-limb joints); cerebral infarction within the preceding 12–24 months (recovery period); MMSE score < 24 points, indicating at least mild dementia and limiting the ability to participate.

Because allocation was not random and group sizes were unequal, chance imbalances in baseline characteristics between groups cannot be excluded. For this reason, the primary analyses focused on within-group pre–post changes, while between-group differences were interpreted descriptively.

### 2.3. Standard Inpatient Rehabilitation

All participants, irrespective of group, received standard inpatient neurorehabilitation in accordance with national clinical guidelines for elderly patients with chronic cerebral ischaemia and frailty. The program included individually tailored combinations of physiotherapy, therapeutic exercises to improve gait and balance, occupational therapy, speech and cognitive training (where indicated), medical optimisation, and education on fall prevention. EMS or sham stimulation, where applicable, was delivered in addition to this routine program and did not replace any scheduled therapy components.

### 2.4. Electrical Muscle Stimulation (EMS) Protocol

The EMS course was designed to span the typical two-week inpatient hospitalisation (two cycles of 5 working days). Patients in the EMS and sham groups underwent from 3 to 9 EMS procedures, depending on length of stay and organisational factors. Each procedure lasted 20 min, with approximately 5 min of stimulation for the anterior and posterior aspects of the thigh and lower leg on each limb.

EMS was delivered using two single-channel Amplipulse-5DS stimulators (SPDS LLC, Yaroslavl, Russia) in sinusoidal alternating current mode with the following parameters: carrier frequency: 5 kHz; burst modulation frequency: 50 Hz; duty cycle: 4 s ON, 6 s OFF (4 s stimulation/6 s rest); session structure: ~20 min total; daily or near-daily sessions on working days.

In the EMS group, large self-adhesive electrodes were placed bilaterally over both the anterior and posterior aspects of the thigh and lower leg of each limb, targeting the major knee and ankle flexor and extensor muscle groups. Electrode positions were kept constant between sessions by marking the skin and using anatomical landmarks. The same electrode montage was used in the sham EMS group (see Supplementary Figure S1 for an illustration). In the EMS group, stimulation amplitude was initially set to 10 mA and then gradually increased to the highest intensity the patient could comfortably tolerate while producing clearly visible, fused contractions in the target muscles. The typical stimulation amplitude ranged from approximately 22 to 24 mA. Patients were encouraged to remain relaxed during stimulation, and short pauses could be added if discomfort or fatigue occurred.

In the sham group, identical electrode placement and timing (5 kHz carrier, 50 Hz burst modulation, 4 s ON/6 s OFF, 20-min duration) were used, but amplitude was set to 4–5 mA, below the motor threshold and without visible muscle contraction, producing only a mild tingling sensation. This was intended to mimic the sensory experience of EMS while avoiding significant neuromuscular activation. Blinding success was not formally assessed in either group, which we acknowledge as a limitation of this study.

### 2.5. Questionnaires and Instrumental Methods

To obtain objective information on the effectiveness of the EMS course for the lower extremities, the following research methods were used before and after the completion of the EMS course in a hospital setting:

1. Functional Mobility Assessment in Elderly Patients [19]: Gait function (0 to 12 points) and balance (0 to 16 points) were assessed separately. The combined score (0 to 28 points) was used to interpret the results—a score of <24 points indicates a risk of falls, while a score of <19 points indicates a high risk of falls.
2. Rivermead Mobility Index [20]: A total of 0 points represents an inability to independently perform any voluntary movements, and 15 points represents the ability to run 10 m.
3. Timed Up and Go Test [21]: Equipment required for the test includes a stable chair with armrests, a tape measure, coloured tape or marker, and a stopwatch. During the test, the patient should wear their usual shoes and use their usual mobility aid (e.g., a cane or walker). Measure a 3-m distance from the chair and mark it with coloured tape or marker so that the mark is visible to the patient. The patient should sit with their back against the back of the chair and their thighs fully touching the seat. The patient is allowed to use the armrests while sitting and when standing. Normally, healthy older adults typically complete the test in 10 s or less. A test time of more than 14 s in home-dwelling elderly patients indicates gait and balance disorders and a risk of falls. Test results correlate with walking speed, balance ability, functional activity level, and ability to go outside.
4. Dynamometry was performed in the supine position using a load cell mounted in a frame under a footplate that registered ankle plantarflexion force. Measurements were taken from the leading leg with the hip, knee, and ankle positioned at approximately 90°. Maximal voluntary force (MVF, kg) was determined as the highest value from three maximal ankle extensions. Similar pedal- or footplate-based dynamometer set-ups are widely used to quantify lower-limb strength and power in clinical and research settings [22,23,24].
5. A postural examination was performed using the BioMera stabilographic platform (BioMera LLC, Moscow, Russia). The patient stood on the platform for one minute with eyes open (EO), and then for another minute with eyes closed (EC). The velocity of the centre of pressure (V, mm/s), the length of the trajectory of the centre-of-pressure movement over 1 min (statokinesiogram length, L, mm), the area of oscillations of the centre of pressure (statokinesiogram area, S, mm^2^), and the Romberg coefficient—a parameter characterising the relationship between the visual and proprioceptive systems—were recorded. The Romberg coefficient is determined as the ratio of the statokinesiogram area during EO to that during EC, expressed as a percentage.
6. Muscle tone of the lower extremities was assessed at rest using a MyotonPRO myotonometer (MyotonLTD, Tallinn, Estonia). The device applies five short mechanical impulses (15 ms) of a stable force (0.4 N) and records the response from the tissue under study, calculating the resistance of biological soft tissues to deformation force (stiffness) [25,26]. The viscoelastic characteristics of the soleus, medial and lateral gastrocnemius, tibialis anterior, semitendinosus, biceps, rectus and vastus lateralis muscles were recorded, as well as those of the Achilles tendon. During this examination, the patient was in the prone or supine position. To standardise the position of the lower extremities, special support was placed under the knee and ankle joints in the prone and supine positions, respectively.

### 2.6. Statistical Analysis

Statistical analysis and graph plotting were performed using GraphPad Prism 8 (GraphPad Software, San Diego, CA, USA). Given the non-randomised observational design, unequal group sizes, and potential baseline imbalances, the primary inferential analyses focused on within-group pre–post changes for each of the three groups separately.

For continuous outcomes (Tinetti scores, Rivermead Mobility Index, TUG time, MVF, stabilographic parameters, and myotonometric stiffness), pre–post differences within each group were analysed using paired tests with a two-sided significance level of α = 0.05 (paired *t*-test or Wilcoxon’s signed-rank test depending on distributional assumptions). Data are presented as mean ± standard deviation (SD) or mean ± standard error of the mean (SEM), as appropriate. Post-intervention analyses were performed per protocol. Participants who discontinued the intervention were excluded from the corresponding post-intervention analyses; missing data were not imputed. Because allocation was not random and the sample size was modest, we did not perform formal between-group hypothesis testing of change scores (e.g., group × time interaction terms). Instead, differences in the magnitude and direction of change between the EMS, sham, and control groups are summarised descriptively (e.g., as “numerically larger” or “smaller” changes), and interpreted as exploratory and hypothesis-generating only.

For bar graphs, mean values with SD are displayed, and individual patient values are overlaid as points, allowing readers to appreciate the distribution of responses.

## 3. Results

A total of 52 inpatients with chronic cerebral ischaemia were enrolled and allocated to three groups (EMS, sham EMS, and no stimulation). Eight patients discontinued the intervention prematurely and were excluded from post-intervention analyses. Baseline demographic characteristics of the three study groups are summarised in Table 1.

This study found that the employed EMS protocol for the lower-leg muscles was well-tolerated by this patient age group, with no significant adverse effects recorded.

Some patients reported moderate muscle soreness for 1–2 days following stimulation at a relatively high amplitude (over 22 mA), comparable to post-exercise soreness, with an intensity of up to 3 points on the 10-point pain scale.

EMS procedures generated interest and positive emotions in patients. All patients reported a subjective positive effect from EMS after 3–4 sessions. Following 8–10 sessions, all patients reported improvements in postural stability and gait, as well as an increased ease of climbing stairs. Specifically, after the EMS course, patients could climb, on average, one more flight of stairs.

No significant changes were observed in the results of any of the methods used in patients in the control and sham groups. However, the efficacy of the EMS protocol was demonstrated in the EMS group.

**Figure 1:**
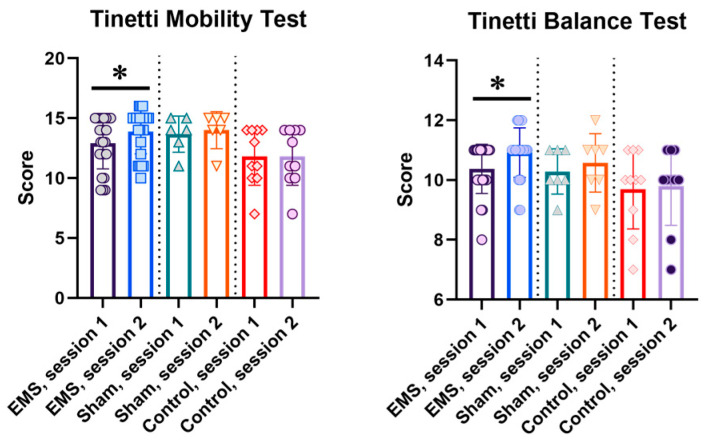
Balance and gait function indicators according to the Tinetti scale in the EMS, control, and sham groups. For the EMS and sham groups, session 1 is before the EMS course, session 2 is after the EMS course; for the control group, sessions 1 and 2 are indicators at the same time intervals as in the experimental groups. *—significant differences, *p* < 0.05.

After the EMS course, an improvement in balance function assessment using the Tinetti scale was noted by 0.96 ± 0.15 points (*p* < 0.05), as was an improvement in gait function using the Tinetti scale by 0.54 ± 0.12 points (*p* < 0.05). Thus, the total Tinetti scale score increased by 1.54 ± 0.23 points (*p* < 0.05) after the EMS course. It is worth noting that, in the EMS group, this parameter was initially below 24 points on average, indicating a risk of falls in the group; however, the EMS course helped to mitigate this risk (Figure 1).

**Figure 2:**
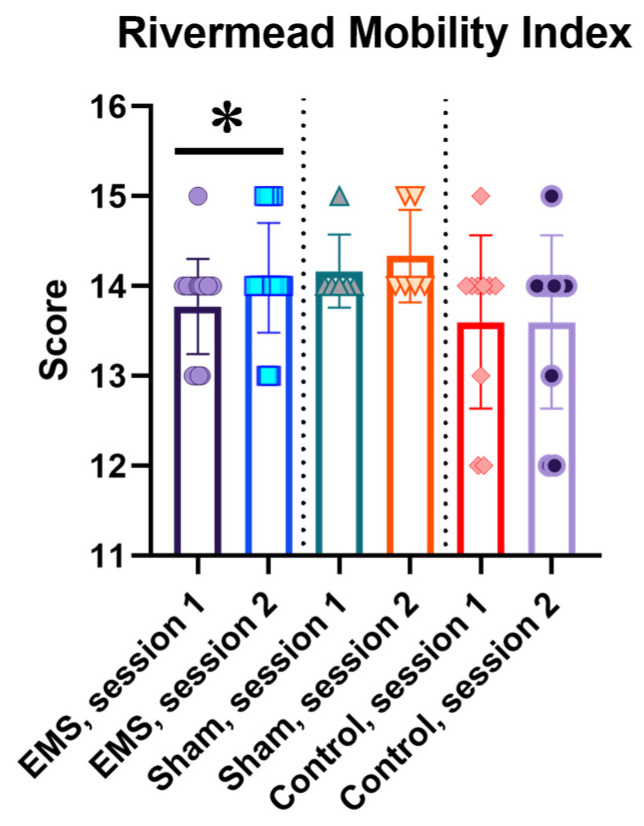
Rivermead Mobility Index in the EMS, control, and sham groups. For the EMS and sham groups, session 1 is before the EMS course, session 2 is after the EMS course; for the control group, sessions 1 and 2 are the indicators at the same time intervals as in the experimental groups. *—significant differences, *p* < 0.05.

The Rivermead Mobility Index also increased after the EMS course by an average of 0.32 ± 0.10 points (*p* < 0.05; Figure 2).

**Figure 3:**
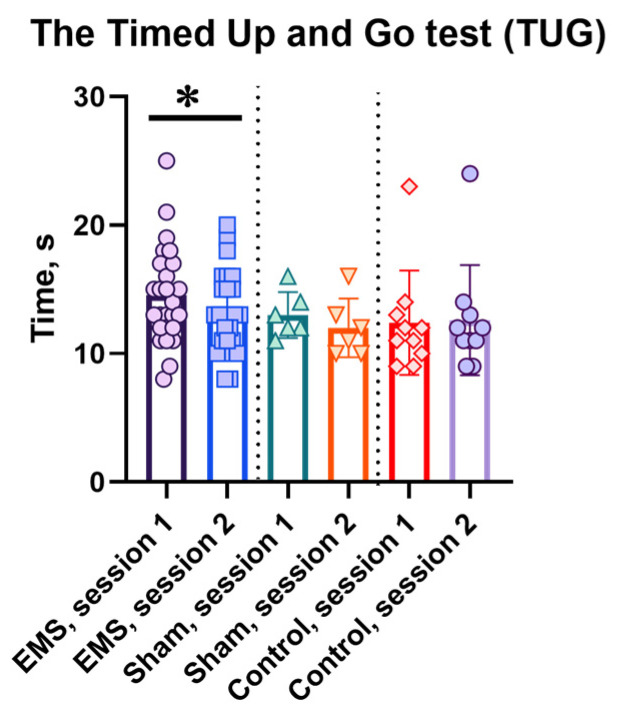
Time to complete the “Stand Up and Go” test in the EMS, sham, and control groups. For the EMS and sham groups, session 1 is before the EMS course, session 2 is after the EMS course; for the control group, sessions 1 and 2 are the indicators at the same time intervals as in the experimental groups. *—significant differences, *p* < 0.05.

The results of the “Stand Up and Go” test are also noteworthy: while before the EMS course, the test completion time was 14.56 ± 3.64 s—indicating the presence of motor impairments and the risk of falls—a decrease in the test completion time after the EMS course to 13.00 ± 3.09 (mean ± SD) demonstrated the elimination of this risk and an increase in patients’ functional capabilities (Figure 3). Thus, after the EMS course, patients completed the “Stand Up and Go” test 1.56 ± 0.35 s faster (*p* < 0.05).

**Figure 4:**
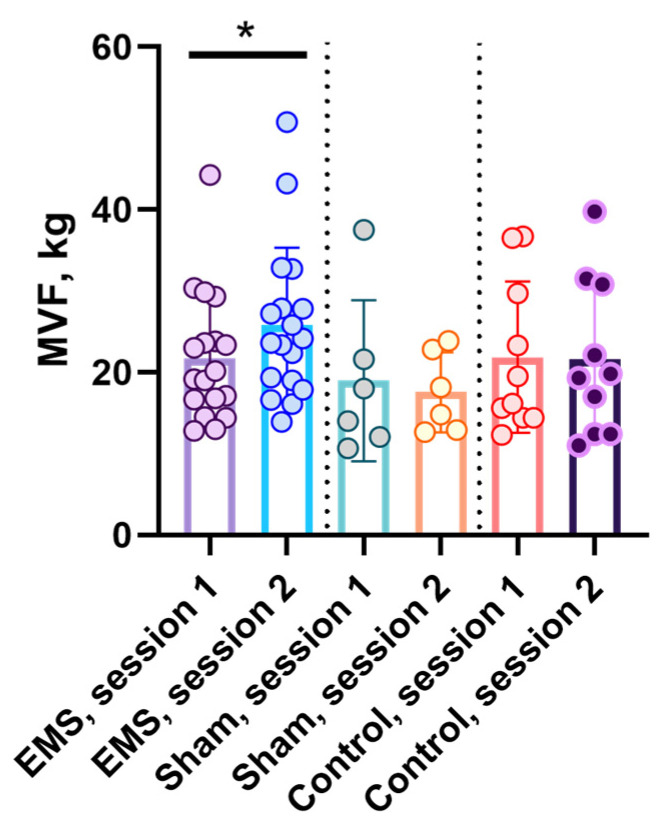
Maximum voluntary ankle extension force in EMS, control, and sham groups. For the EMS and sham groups, session 1 is before the EMS course, session 2 is after the EMS course; for the control group, sessions 1 and 2 are the indicators at the same time intervals as in the experimental groups. *—significant differences, *p* < 0.05.

After the EMS course, MVF in the EMS group significantly increased during ankle extension by 19.3% (*p* < 0.05, Figure 4). In other groups, no significant changes were observed: in the control group, a slight increase in MVF of 7.1% was recorded, and in the sham group, a decrease of 8.24%.

**Figure 5:**
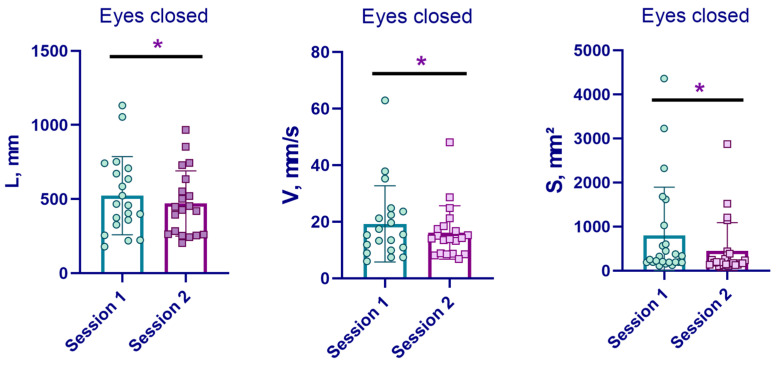
Postural test results in the EMS group before (session 1) and after (session 2) the stimulation course. *—significant differences, *p* < 0.05.

After the EMS course, patients demonstrated a more confident vertical stance; however, reliable improvements in stabilometric parameters were recorded only in the eyes-closed test (EC, Figure 5). Thus, with EC, a reliable decrease in the statokinesiogram length (L, mm) from 669.8 ± 109.2 to 504.2 ± 83.47 mm (*p* < 0.05) was recorded in 15 patients, as well as a decrease in the speed of movement of the centre of pressure (V, mm/s) from 22.36 ± 3.646 to 16.86 ± 2.783 mm/s (*p* < 0.05). At the same time, the statokinesiogram area (S, mm^2^) in all patients in the EMS group significantly decreased from 730.5 ± 202.9 to 413.6 ± 117.8 mm^2^ (*p* < 0.05). In patients of the control and sham groups, no significant changes in postural stability indicators were observed.

**Figure 6:**
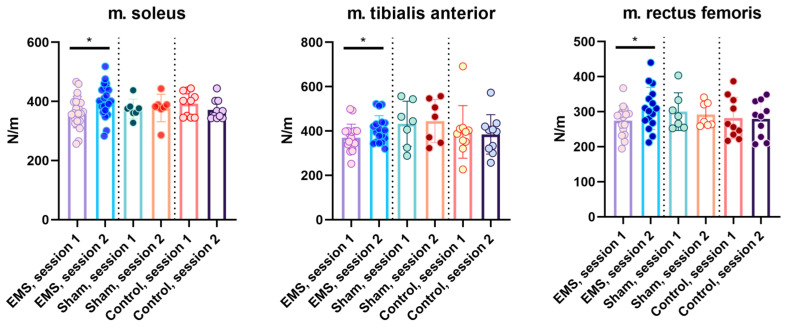
Stiffness in the EMS, control, and sham groups. For the EMS and sham groups, session 1 occurred before the EMS course, session 2 occurred after the EMS course; for the control group, sessions 1 and 2 were performed at the same time intervals as in the experimental groups. *—significant differences, *p* < 0.05.

A study of muscle tone in the stimulation group before and after EMS showed significant changes in the Stiffness parameter in the soleus, tibialis anterior, and rectus femoris muscles. In m. soleus, transverse stiffness significantly increased by 11% from 354.6 ± 6.2 to 370.8 ± 7.6 N/m (*p* < 0.05). In m. tibialis anterior, it increased by 10% from 363.0 ± 11.4 to 400.9 ± 13.8 N/m (*p* < 0.05). In the m. rectus femoris, stiffness increased by 12.7% from 276.1 ± 9.4 to 295.6 ± 13.1 N/m (*p* < 0.05). In the control and sham groups, no significant changes in muscle tone were registered (Figure 6).

Further studies should aim to enrol larger participant cohorts and investigate the effectiveness of other EMS protocols in elderly patients with impaired motor function.

## 4. Discussion

### 4.1. Principal Findings and Interpretation

Adding a clearly described Russian-current electrical muscle stimulation (EMS) protocol to usual inpatient neurorehabilitation for older adults with chronic cerebral ischaemia proved to be feasible, well-tolerated, and associated with meaningful within-group functional gains. The absence of SAEs and the low rate of minor skin events align with the safety profiles reported in RC studies [9]. In the EMS group, we observed statistically significant pre–post improvements in balance and gait (Tinetti total and subscale scores, TUG), mobility (Rivermead Mobility Index), ankle extensor strength (MVF) and postural control under eyes-closed conditions (reduced statokinesiogram path length, mean COP velocity and sway area), along with favourable changes in muscle mechanical properties on myotonometry. In contrast, changes over the same period in the sham and standard-care-only groups were smaller and mostly non-significant. Thus, within the constraints of this non-randomised observational design, our findings suggest that integrating Russian-current EMS into routine inpatient rehabilitation may be associated with the most pronounced within-group functional gains in elderly patients with chronic cerebral ischaemia. However, because allocation was not random and our inferential analyses focused on within-group pre–post changes rather than formal between-group hypothesis testing, apparent differences in the magnitude of improvement across the three groups should be interpreted as descriptive rather than as definitive evidence of superiority.

The pattern of stabilographic change is of particular interest: significant reductions in path length, COP velocity, and sway area were observed only under eyes-closed conditions. This profile is compatible with improved postural control when visual input is removed and may reflect better use of ankle strategy or enhanced confidence in stance. Nevertheless, no dedicated sensory-balance tasks were included.

### 4.2. Placement Within the RC Literature

Our results are consistent with the pilot RCT (randomised controlled trial) demonstrating that quadriceps RC improves 10-m walk speed in chronic stroke survivors without SAEs [9]. They also cohere with systematic reviews showing that EMS (across waveforms) improves gait and balance when embedded in structured programs—suggesting that well-specified RC can serve as a tolerable delivery mode of EMS in older adults [7,8]. At the same time, laboratory and crossover studies in healthy adults underscore that RC effectiveness depends on parameterisation—burst frequency, duty cycle, ON/OFF timing and ramping influence torque production, neuromuscular efficiency, and perceived discomfort, while precise motor-point placement affects torque yield for a given current. Such parameter sensitivity likely explains some of the heterogeneity across published RC studies and emphasises the value of our explicit reporting of carrier frequency, burst architecture, duty cycle, session structure, electrode montage, and amplitude range. By providing detailing electrode placement, the present study contributes to the reproducibility of RC protocols in geriatric neurorehabilitation settings.

### 4.3. Clinical Significance of Change Magnitudes

In frail inpatients with chronic cerebral ischaemia, even small improvements across multiple domains may still be valuable as part of a multimodal rehabilitation approach, but larger trials explicitly powered on MCID-based endpoints are needed to clarify the clinical relevance of these changes.

### 4.4. Safety, Tolerability, and Implementation

With no SAEs, manageable discomfort through intensity titration, and short ON periods, our implementation mirrors the benign safety profile reported in the contemporary RC literature [9]. Routine skin inspection, stop rules, and documentation of delivered mA and %MTC are practical safeguards for frail inpatients, and they facilitate future dose–response modelling. For clinical adoption, we recommend (I) motor-point mapping (e.g., using atlas coordinates) [16], (II) progression to the highest comfortable contraction, and (III) task integration (e.g., sit-to-stand, supported overground walking) to reinforce functional synergies during stimulation.

From an implementation perspective, explicit documentation of electrode placement using motor-point maps, progression to the highest comfortable contraction, and integration of stimulation with behavioural goals (e.g., standing balance and overground walking) may enhance functional carry-over. The fact that many patients reported increased confidence and improved stair climbing after 3–10 sessions suggests that EMS may have motivational as well as neuromuscular benefits in this setting, although such patient-reported outcomes were not formally quantified in the present study.

### 4.5. Implications and Next Steps

For older adults with weak knee extensors and impaired ankle strategy, RC offers a tolerable, reproducible means of eliciting visible, task-relevant contractions within constrained therapy time. Priority directions are (I) a multi-centre RCT powered for functional co-primary endpoints (e.g., TUG and gait speed) with falls as a key secondary outcome over 3–6 months; (II) head-to-head benchmarking of RC against optimised pulsed protocols under harmonised reporting (device model, burst architecture, duty/ON–OFF timing, ramping, motor-point coordinates, delivered mA, and %MTC); and (III) dose–response modelling of intensity, session number, and duty to define minimal effective and optimal regimens. Transparent protocol appendices will improve reproducibility and accelerate synthesis.

### 4.6. Strengths and Limitations

Strengths of this study include a fully specified Russian-current EMS protocol (carrier frequency, burst parameters, duty cycle, session structure, and intensity titration), standardised electrode placement based on a motor-point atlas, blinded outcome assessment, and the combination of validated functional scales with instrumented stabilography and myotonometry. These features enhance reproducibility and allow for comparison with other EMS studies.

At the same time, important limitations must be acknowledged. Allocation to EMS, sham, and control groups was non-random and reflected routine clinical decision-making, leading to unequal group sizes (27 vs. 10 vs. 7) and potential baseline imbalances that we could not fully control. The modest sample size and short follow-up mean that estimates of effect are imprecise and durability beyond the inpatient stay is unknown. This study did not include formal head-to-head comparisons with optimised low-frequency pulsed EMS protocols, so the relative position of Russian-current EMS within the broader EMS “toolbox” remains to be established.

Several further limitations should be noted. First, this was a single-centre study conducted in a specific inpatient neurorehabilitation setting, which may limit generalisability to other healthcare systems and rehabilitation models. Second, outcomes were assessed only over a short interval of approximately 2 weeks; we did not collect long-term follow-up data on functional status or falls, so we cannot determine whether the observed short-term changes translate into sustained benefits or reduced fall incidence. Third, although sham stimulation was designed to mimic active EMS at a sensory level, we did not formally assess blinding success or patients’ beliefs about group allocation, which may have influenced perceived benefits. Finally, as discussed above, this study was not powered or designed for definitive between-group comparisons; the primary inferences are within-group, and any apparent differences in change between EMS, sham, and control groups should be interpreted as exploratory.

## 5. Conclusions

In this prospective single-centre controlled observational pilot of older adults with chronic cerebral ischaemia, inpatient Russian-current electrical muscle stimulation (EMS; 5 kHz carrier, 50 Hz bursts, 4 s ON/6 s OFF) integrated into standard rehabilitation was feasible, well-tolerated, and associated with statistically significant within-group improvements in balance, mobility, ankle extensor strength, and postural stability over approximately 2 weeks. Gains were most consistent for the Tinetti balance and gait subscales, Timed Up and Go performance, and stabilographic measures under eyes-closed conditions, with complementary changes in muscle stiffness on myotonometry. In contrast, sham and standard-care-only groups showed only small, non-significant changes across the same outcomes. While the design minimised assessor bias, the non-random allocation, modest sample size, focus on within-group pre–post analyses, and short follow-up limit inference on durability and generalisability. Nevertheless, the detailed reporting of stimulation parameters, electrode placement, and safety monitoring in this study provides a replicable framework for Russian-current EMS as an adjunct to inpatient rehabilitation and supports further investigation of its role in geriatric neurorehabilitation.

## Figures and Tables

**Table 1:** Baseline demographic characteristics of the three study groups.

Characteristic	Stimulation (n = 27)	Sham (n = 7)	No Stimulation (n = 10)
Female, n (%)	22 (81.5%)	7 (100.0%)	9 (90.0%)
Male, n (%)	5 (18.5%)	0 (0.0%)	1 (10.0%)
Age (years)	73.0 ± 7.3	74.1 ± 7.0	79.0 ± 7.0
Height (cm)	163.4 ± 8.7	155.6 ± 5.3	156.6 ± 7.7
Weight (kg)	75.7 ± 17.0	68.4 ± 14.4	74.1 ± 8.4
Mini-Mental State Examination (MMSE) score	27.6 ± 1.5	27.3 ± 1.1	26.7 ± 1.2

Data are expressed as mean ± standard deviation (SD) for continuous variables and as number (percentage) for categorical variables.

## Data Availability

Original data are available from the corresponding author on reasonable request, subsequent approval, and completion of a legal data-sharing agreement. The data are not publicly available due to local ethics requirements. De-identified data will be made available after documentation, as appropriate, when completed by the relevant entities.

## Supplementary Materials

The following supporting information can be downloaded at https://www.mdpi.com/article/10.3390/life16010126/s1. Figure S1. Electrostimulation protocol. Figure S2. Schematic of electrode placement and stimulation sequence for the lower-limb segments (1–4). Figure S3. Schematic of stimulation. Stimulation sequence for the lower-limb segments (1–4). Figure S4. Dynamometry was carried out in the supine position using a load cell mounted in a frame under the plate that took the loading from ankle extension (plantar flexion). Figure S5. Posturological examination on the BioMera stabilographic platform. Figure S6. Measurement of viscoelastic properties of the soleus, tibialis anterior, and rectus femoris muscles. Table S1. STROBE statement—checklist.

## Abbreviations

The following abbreviations are used in this manuscript:

CCI	Chronic cerebral ischaemia
COP	Centre of pressure
EMS	Electrical muscle stimulation; electromyostimulation
MMSE	Mini-Mental State Examination
MVF	Maximal voluntary force
RC	Russian current
RCT	Randomised controlled trial
SAE	Serious adverse event
TUG	Timed Up and Go
%MTC	Percent of maximally tolerated current
L	The length of the trajectory of the centre of pressure movement over 1 min, mm
V	Mean velocity of the centre of pressure, mm/s
S	Statokinesiogram area, mm^2^
