# Supplementary material for: Electrical Muscle Stimulation with Russian Current in Chronic Cerebral Ischaemia
Source: Life (Basel). 2026 Jan 14;16(1):126. doi: 10.3390/life16010126 (PMC12843009; doi:10.3390/life16010126)

Supplementary Materials

Table S1. STROBE statement—checklist

Section / Topic	Item	Checklist item	Location in article
TITLE and ABSTRACT	1a	Indicate the study design using a commonly used term in the title or abstract.	Abstract: "Prospective single-centre controlled observational pilot…"
	1b	Provide an informative and balanced summary of what was performed and what was found.	Structured Abstract (Objective, Design, Methods, Results, Conclusions).
INTRODUCTION	2 (Background)	Explain the scientific background and rationale for the investigation being reported.	Introduction Section, paragraphs 1–4 (aging, CCI, EMS/RC rationale).
	3 (Objectives)	State specific objectives or hypotheses.	Last paragraph of Introduction Section ("The primary aim… We hypothesised that…").
METHODS	4 (Study design)	Present key elements of study design early in the paper.	Methods Section—"study design" (prospective single-centre controlled observational pilot study).
	5 (Setting)	Describe the setting, locations, and relevant dates, including periods of recruitment and follow-up.	"Study design" and "Participants" subsections: inpatient neurorehabilitation centre; Moscow; Sept 2021–Oct 2024; ~2-week inpatient stay.
	6a (Participants)	Give eligibility criteria and the sources and methods of selection of participants. Describe methods of follow-up.	Methods Section—"Participants" (inclusion/exclusion criteria, ICD-10 I67.8 diagnosis, reasons for non-participation and drop-out)
	6b	For matched studies, give matching criteria and number of exposed/unexposed.	Not applicable (no matching).
	7 (Variables)	Clearly define all outcomes, exposures, predictors, potential confounders, and effect modifiers.	"Study design", "Standard inpatient rehabilitation", "Electrical muscle stimulation (EMS) protocol", "Questionnaires and instrumental methods" (Tinetti, RMI, TUG, MVF, stabilography, myotonometry; exposure = EMS / sham / standard care).
	8 (Data sources/measurement)	For each variable of interest, give sources of	"Questionnaires and instrumental methods" (detailed procedures for each

		data and details of assessment methods.	test; device and measurement conditions).
	9 (Bias)	Describe any efforts to address potential sources of bias.	“Study design” (acknowledges non-random allocation and unequal group sizes); “statistical analysis” (focuses on within-group changes); “strengths and limitations” (discussion of selection and confounding).
	10 (Study size)	Explain how the study size was arrived at.	Currently implicit as a pilot (n=52 admitted, 44 analysed).
	11 (Quantitative variables)	Explain how quantitative variables were handled in the analyses (e.g., groupings, transformations).	“Statistical analysis” (continuous outcomes; paired tests; presentation as mean±SD/SEM; no categorisation of continuous variables).
	12a (Statistical methods)	Describe all statistical methods, including those used to control for confounding.	Methods Section—“Statistical analysis” (within-group pre–post tests, no formal between-group hypothesis testing; per-protocol analyses; missing data not imputed).
	12b	Describe any methods used to examine subgroups and interactions.	Not specifically performed; can mark “Not applicable; no subgroup/interactions planned.”
	12c	Explain how missing data were addressed.	Missing post-intervention data from dropouts were handled per the protocol: participants who discontinued the intervention were excluded from the corresponding post-intervention analyses, and missing data were not imputed. This is described in the “Statistical analysis” subsection (Methods Section, paragraph 2, final sentences) and in the first paragraph of the Results Section (“Eight patients discontinued the intervention prematurely…”).
	12d	If applicable, explain how loss to follow-up was addressed.	“Participants” / “Results” Section
	12e	Describe any sensitivity analyses.	Not performed (exploratory pilot).

RESULTS	13a (Participants)	Report the numbers of individuals at each stage of the study (e.g., eligible, included, completing follow-up, analysed).	Methods Section—“Participants” (numbers enrolled and allocated to three groups); Results Section—first paragraph (numbers analysed and drop-outs).
	13b	Give reasons for non-participation at each stage.	Non-participation was minimal. A total of fifty-two in-patients with chronic cerebral ischaemia were enrolled; eight patients discontinued the intervention prematurely and were excluded from post-intervention analyses. Reasons for discontinuation were not systematically analysed and are therefore not reported in detail. (Methods Section—“Participants”; Results Section—first paragraph).
	13c	Consider use of a flow diagram.	Participant flow (52 enrolled, 8 discontinued, 44 included in post-intervention analyses) is described narratively in Methods Section (“Participants”) and in the first paragraph of the Results Section. No separate flow diagram was prepared.
	14a (Descriptive data)	Give characteristics of study participants (e.g., demographic, clinical, baseline).	Table 1 (baseline characteristics); Methods Section—Participants; Results Section—first paragraph.
	14b	Indicate number of participants with missing data for each variable of interest.	For primary pre–post outcomes, analyses were restricted to participants with complete data; patients who discontinued the intervention (n = 8) were excluded from post-intervention analyses, and no imputation of missing values was performed. Item-level missing data for baseline characteristics were minimal and are not tabulated separately. (Methods Section—“Statistical analysis”; Results Section—first paragraph; Table 1).
	14c	Summarise follow-up time, if appropriate.	Methods Section (“two-week inpatient hospitalisation”, 3–9 sessions).

	15 (Outcome data)	Report numbers of outcome events or summary measures over time.	Results Section: detailed pre–post changes for Tinetti, RMI, TUG, MVF, stabilography (EC), myotonometry; Figures 1–6.
	16a (Main results)	Give unadjusted estimates and, if applicable, confounder-adjusted estimates and precision (e.g., 95% CI).	Results Section: mean differences ±SE/SD with p-values for within-group changes.
	16b	Report category boundaries when continuous variables were categorised.	Not applicable (continuous outcomes kept continuous).
	16c	If relevant, translate estimates of relative risk into absolute risk over a meaningful time period.	Not applicable (no risks/ratios).
	17 (Other analyses)	Report other analyses performed—e.g., subgroup analyses, interactions, sensitivity analyses.	Not performed; can mark "Not applicable".
DISCUSSION	18 (Key results)	Summarise key results with reference to study objectives.	Discussion Section, subsection "Principal findings and interpretation"; first paragraphs of Discussion Section.
	19 (Limitations)	Discuss limitations, addressing sources of potential bias or imprecision, and their direction and magnitude.	Discussion Section, paragraph "Limitations" (non-randomised allocation, small sample size, group-size imbalance, residual confounding).
	20 (Interpretation)	Provide a cautious overall interpretation considering objectives, limitations, multiplicity of analyses, results of similar studies, and other evidence.	Discussion Section: "Placement within the RC literature", "Clinical significance…", "Implications and next steps".
	21 (Generalisability)	Discuss the generalisability (external validity) of the study results.	"Strengths and limitations" / "Implications and next steps" (single-centre, specific setting; need multi-centre RCT).
OTHER INFORMATION	22 (Funding)	Give the source of funding and the role of the funders.	Sections "Funding" (Program of applied research of IBMP RAS…), "Conflicts of Interest".

Figure S1. Electrostimulation protocol. The diagram shows a general view of the "Amplipulse-5DS" electromyostimulator and its output signal, electrode placement, and the sequence of stimulation for the lower-limb segments.

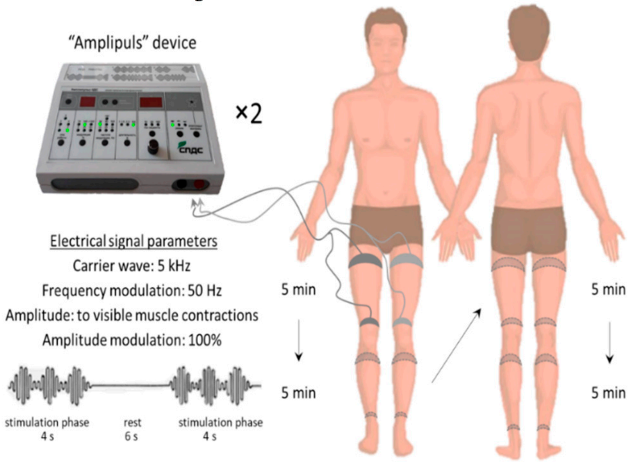

Figure S2. Schematic of electrode placement and stimulation sequence for the lower-limb segments (1-4). The group mean stimulation amplitude for the EMS group is shown for each segment.

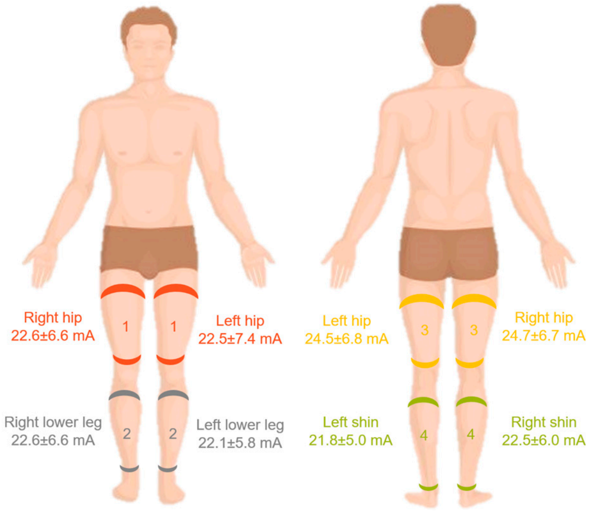

Figure S3. Schematic of stimulation. Stimulation sequence for the lower-limb segments (1-4). The average stimulation amplitude for the sham group is indicated next to each segment.

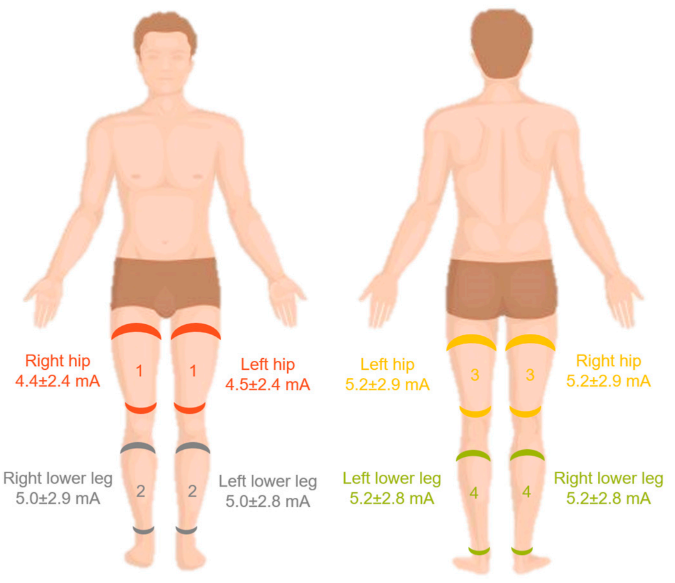

Figure S4. Dynamometry was carried out in the supine position using a load cell mounted in a frame under the plate that took the loading from ankle extension (plantar flexion). The measurement was taken from the leading leg at an angle of 90o at the hip, knee, and ankle joints.

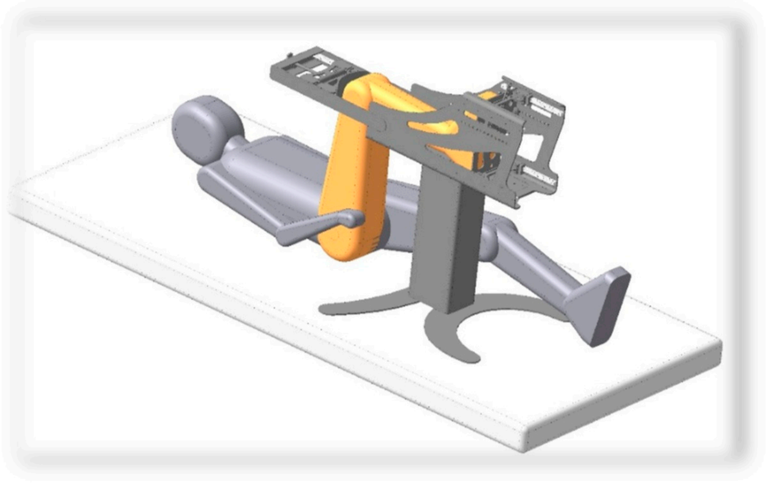

Figure S5. Posturological examination on the BioMera stabilographic platform (BioMera LLC, Russia).

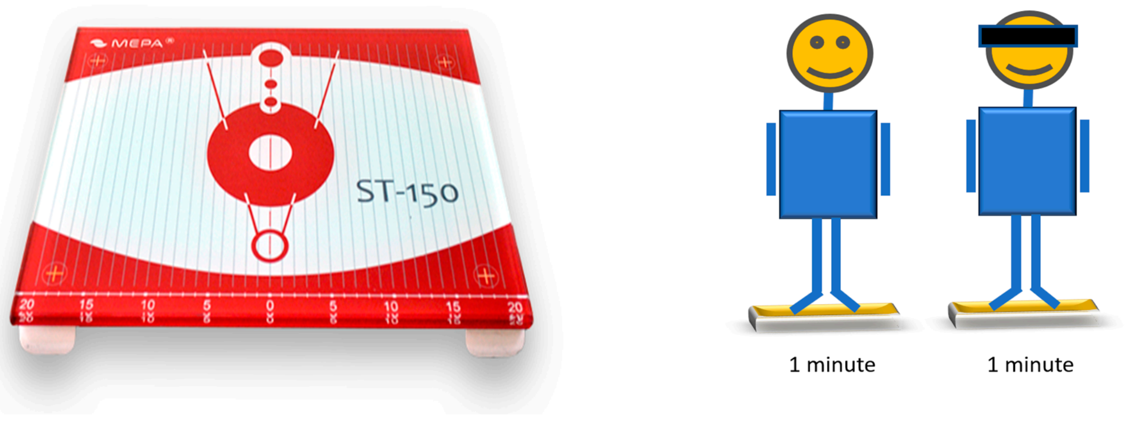

Figure S6. Measurement of viscoelastic properties of the soleus, tibialis anterior, and rectus femoris muscles. Photos from the following: https://www.myoton.com/applications.

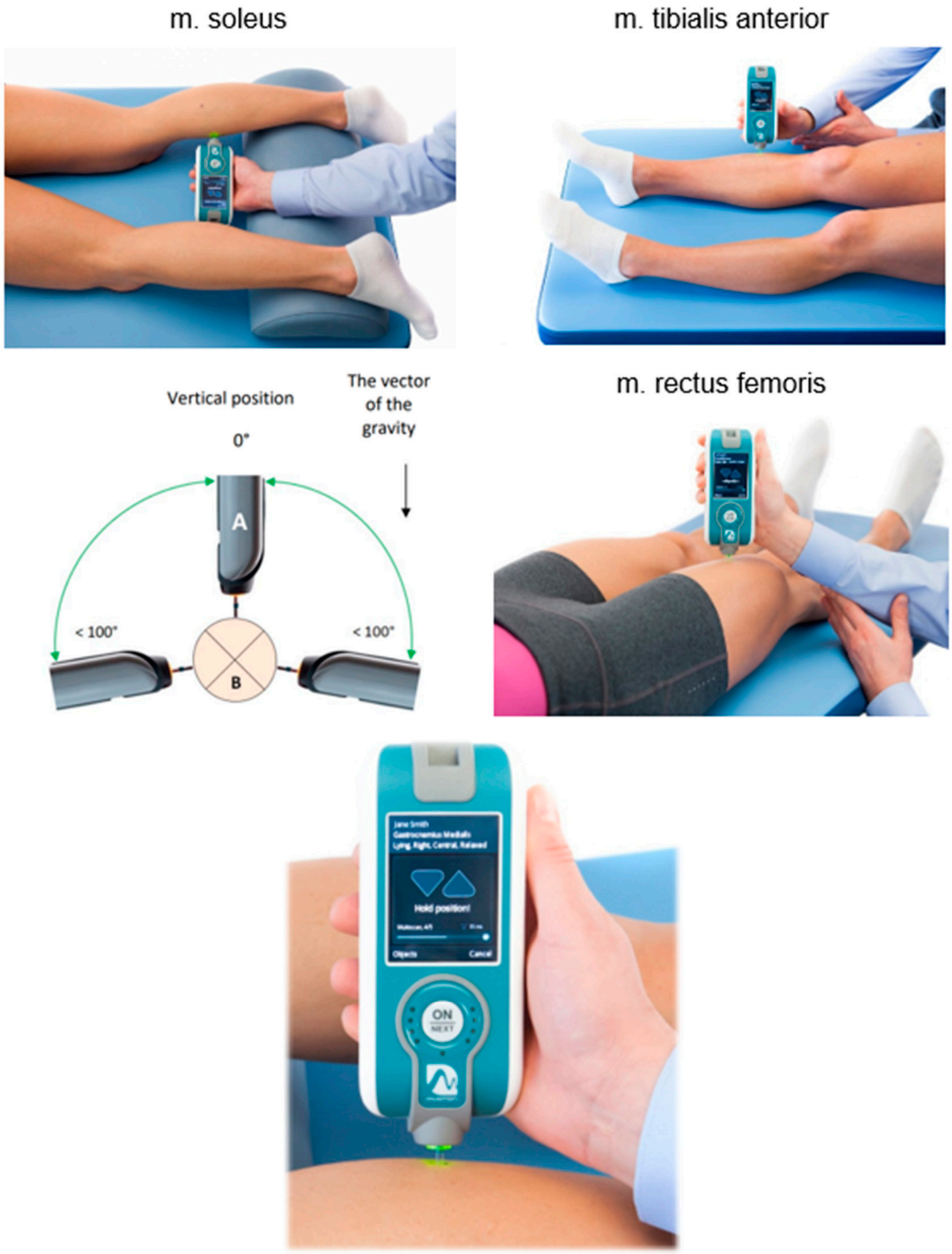

Supplement: Supplementary file 1 [file life-16-00126-s001.zip › life-4018688-supplementary.pdf]
